# Pupil dynamics reveal the tuning of tortricid moths to diel activity

**DOI:** 10.1007/s00359-025-01759-0

**Published:** 2025-10-09

**Authors:** Alejandro Martín-Gabarrella, César Gemeno, Aleš Škorjanc, Andrej Meglič, Gregor Belušič

**Affiliations:** 1 University of Lleida-Agrotecnio-CERCA Center , 25198 Lleida, Spain; 2 Grapevine and Wine Research Center (ICVV) , CSIC-UR-GR , 26007 Logroño, Spain; 3 Biotechnical Faculty , University of Ljubljana , Večna pot 111, 1000 Ljubljana, Slovenia; 4 Eye Hospital , University Medical Centre , Grablovičeva 46, 1000 Ljubljana, Slovenia

**Keywords:** Tortricidae, Pupil, Light adaptation, Circadian rhythm, Temperature

## Abstract

Insect compound eyes adapt in response to average stimulus intensity, but the adaptation is mediated also by intrinsic circadian rhythms and abiotic factors like temperature, which are indirectly related to the stimulus. We studied the effect of light, circadian rhythm, temperature and body size on light adaptation in *Grapholita molesta* (GM), *Lobesia botrana* (LB) and *Cydia pomonella* (CP) (Lepidoptera: Tortricidae). These moths have superposition compound eyes with identical trichromatic photoreceptor sets and temporal resolution; however, the adults are sexually active before (GM), during (LB) or after (CP) sunset, and experience very different light intensity and temperature variations during their activity period. Their eyes were examined with infrared pupillometry, light microscopy and micro-CT. The compound eyes are very small to medium-sized, with a clearly visible superposition pupil. The pupil reflectance of adults entrained to a 12/12 h light/dark cycle for 2 days maintained the entrained rhythm for days, closing and opening during the subjective day and night, respectively. Circadian rhythm was the most robust in CP. A fully open pupil was forced to close with a light pulse, and pupil brightness changes were monitored at 15 and 22 °C. The experiment revealed complex and different pupil dynamics among species at both temperatures. GM, the smallest species, was most affected by the lower temperature. The experimental paradigm can be used for high-throughput, non-invasive monitoring of multiple species’ response to climate change, and to chemical and light pollution.

**Supplementary Information:**

The online version contains supplementary material available at 10.1007/s00359-025-01759-0.

## Introduction

Animal eyes have evolved to cope with a vast range of illumination levels across different habitats, with daily changes in absolute illumination, light spectra and temperature (Warrant and Somanathan 2022). Therefore, many species have eyes that are tightly tuned to their visual ecologies (Lebhart and Desplan 2017). Closely related species that occupy different ecological niches are expected to possess eyes with subtle adaptations to their visual environments. Arguably, the largest selective pressure on the visual system is the amount of available light, or its variation between day and night. Evolutionary adaptations to low light at night encompass increased light sensitivity due to eye optics (Stöckl et al. 2022; Frederiksen and Warrant 2008), tuning of spectral sensitivity (Akiyama et al. 2022), and neural networks capable of photoreceptor signal pooling (Stöckl et al. 2016). In many animals, nocturnal activity is also associated with a low body temperature, compared to daytime, which slows physiological processes including those related to vision. Among insects, larger moths have evolved limited endothermy to cope with the cold (Heinrich 1987). In smaller moths like Tortricidae, endothermy has not been described yet, probably due to the unfavourable thermal losses associated with their large surface-to-volume ratio. Interestingly, among the species studied here, nocturnal and diurnal activity is associated with the largest and smallest body size, respectively. We hypothesize that to tolerate cold, tortricid moths evolved motor proteins and enzymes that remain faster and more efficient at low temperatures. To reveal these adaptations, we studied the pupillary reflex in three closely related moths from the family Tortricidae, which are active either during the day, night or at sunset.

Insects possess compound eyes composed of discrete units called ommatidia. Most insects have apposition compound eyes, in which individual ommatidia are optically isolated. Although these eyes typically exhibit low light sensitivity and are generally adapted for diurnal vision, some species—such as certain nocturnal bees—have evolved adaptations that allow vision under dim-light conditions (Warrant 2008). Generally, night-active insects have superposition compound eyes, where the dioptrical components and the light-sensitive photoreceptor cells, the rhabdomeres, are separated by a translucent gap called ‘the clear zone’ (Kunze 1979). In superposition eyes, each rhabdomere receives light from many adjacent facets, thus, increasing light sensitivity sufficiently to allow vision in dim light. The amount of light is controlled by a powerful pupillary mechanism, created by dark pigment granules located in specialized pigment cells that absorb the superposed light rays (Land and Nilsson 2012). In superposition eyes with so-called ‘longitudinal pupil’, the pupillary pigments in the secondary pigment cells migrate between the two extreme positions along the clear zone, distal and proximal (Höglund 1963). This mechanism is found in many moths, including the relatively large Noctuid *Agrotis* sp. (Tuurala 1954). In eyes with ‘cone pupil’, pigments in the primary pigment cells migrate only around the crystalline cone tip (Warrant and McIntyre 1996). This mechanism mediates pupil dynamics in Hesperiid butterflies (Horridge et al. 1972). Finally, a mixed mechanism called ‘dual pigment migration’, mediated by the pigment granules migrating in the primary and secondary pigment cells, is frequently found in small nocturnal moths (Warrant and McIntyre 1996), for example, in the Pyralid *Ephestia spp.* (Horridge and Giddings 1971). Tortricid moths have compound eyes with superposition optics and a longitudinal pupil (Satoh et al. 2017; Yang et al. 2024). Pupil dynamics in superposition eyes should depend on the speed of the motor proteins that move the pigment granules and on the thickness of the clear zone; smaller eyes could in principle adapt faster. At different temperatures, pupil speed will be also affected by the thermal adaptations of the molecular apparatus mediating granule migration.

Superposition pupil is much slower than the pupil in apposition eyes of diurnal insects (Warrant and McIntyre 1996; Bernard and Wehner 1980; Nordtug and Melo 1992) meaning that the moths are inherently less capable of adapting to sudden changes in light intensity (Dreisig 1981). To better cope during the activity period, the superposition pupil might be pre-set by feed-forward mechanisms, driven by the intrinsic circadian rhythms (Nordstrom and Warrant 2000) and fine adjusted by the feed-back mechanism of acute light adaptation. Thus, the adaptation state in nocturnal and crepuscular species might be set in advance by the interplay of circadian rhythm and the predictable temperature gradient between day and night, while diurnal species that are active in less variable temperature and light conditions should rely more on the adaptation to the current average amount of light.

In superposition eyes the incident light not absorbed by photoreceptor cells is reflected from the tracheolar tapetum located at the base of the retina, and scattered from the rhabdom layer back to the dioptrical apparatus. In a dark-adapted eye, this reflection is seen by the observer as a bright circular spot at the centre of the eye, the so-called superposition pupil (not anatomically equivalent to the human pupil). Pupil brightness diminishes with light adaptation and can be used as a proxy for the evaluation of the light adaptation dynamics in superposition eyes (Dreisig 1981). Near-infrared (NIR) light allows monitoring of pupil diameter without triggering phototransduction, as it is reflected by the tapetum and partially blocked by screening pigment granules. The reflection intensity indicates the distance of the pigment from the rhabdom, thus reflecting pupil openness (Stavenga 1979).

Here we compared three tortricid moth species that are active during the day, dusk and night, respectively: the oriental fruit moth *Grapholita molesta* (Busck), the European grapevine moth *Lobesia botrana* (Dennis and Schiffermüller), and the codling moth *Cydia pomonella* L. (from here on, GM, LB and CP, respectively). We have previously found that these species did not differ in their spectral sensitivity and flicker fusion frequency (Martín-Gabarrella et al. 2023). Next, in order to reveal any putative species-specific adaptation of their visual systems to their different ecological niches, we study light adaptation by observing pupil dynamics. Intrinsic and evoked pupil dynamics was monitored with an USB microscope and NIR illumination at two different temperatures, emulating day and night conditions. To interpret any differences among species, we measured eye size and clear zone thickness using light microscopy and micro-CT imaging. We hypothesize that pupil response time is primarily a function of eye diameter and temperature. Three phylogenetically related species with different size and eye size are tested at different temperatures to check this claim.

## Materials and methods

### Insects

Larvae were reared on a semi-artificial diet (Ivaldi-Sender 1974) under a 16:8 L: D photoregime at 25 ± 1 °C. Pupae were shipped from Spain to Slovenia and kept at 22 ± 1 °C in incomplete darkness, interrupted by brief daily incubator inspections. Newly emerged adults were separated from pupae every other day, being 0- to 2-days old at the separation day, 1- to 3-days old on the following day, and so on. They were kept under a 12:12 L: D photoregime at either 15 ± 1 °C or 22 ± 1 °C (low and high temperature regimes). Adults had unrestricted access to 10% sucrose in water until tested.

### Eye morphology

Morphological aspects of the eye were observed using several optical methods. Computed tomography scan (CT-scan) was performed in 1 individual of each sex and species to describe ommatidia number and width, inter-ommatidial angle (estimated by taking a cross-section perpendicular to the eye surface, counting the number of ommatidia along the arc, and dividing 180° by this number), eye radius (distance from the geometrical centre of the idealized eye sphere to its perimeter), eye width (in both, dorso-ventral and antero-posterior aspects), and clear-zone length. Bright field microscopy of thin sections of one individual of each species provided finer structural detail than the CT-scan and a better estimation of the clear-zone length. Finally, external measurements of the eye width (in the dorso-ventral and antero-posterior aspects) were obtained from 10 individuals of each sex and species with a stereo microscope.

#### Micro-computed tomography (micro-CT)

Heads from light-adapted insects were isolated under bright preparation light, fixed in 70% ethanol for a week and then stained with 1% phosphotungstic acid (prepared in 70% ethanol) for another week. After staining the heads were washed in 70% ethanol and placed into small plastic tubes filled with 70% ethanol for imaging. Scanning was performed with a Neoscan N80 microtomograph (Neoscan, Mechelen, Belgium) at 50–55 kV and 80–100 µA, with a resolution of 0.5–0.6 μm. Scans were acquired with a rotation step of 0.15° over 180°, averaging three images per step. Virtual cross sections were reconstructed using Neoscan 80 software (version 2.2.4), and imported into Dragonfly software (version 2021.1.0.977) (ORS Inc., Mississauga, Ontario, Canada) for 3D reconstruction and visualization of sections in arbitrary planes. Longitudinal sections along the dorso-ventral eye axis were calibrated and exported as graphic files to CorelDRAW 14.0 (Alludo, USA), where the eye radius was estimated by manually fitting a circle to the eye’s outer edge in the region of maximal eye diameter.

#### Light microscopy

Heads from light-adapted males of the three species were hemisected under bright light, fixed in 3.5% glutaraldehyde and 4% paraformaldehyde at 4 °C, dehydrated in ethanol series (50−100% in 10% increments), and embedded in Spurr’s resin (Structure probe Inc., USA) using a gradual infiltration process over two days. Semi-thin (1 µm) sections were obtained using an ultramicrotome (Ultracut, Leica, Germany) and stained with Richardson’s blue stain before imaging with an Axioskop microscope (Zeiss, Germany).

### Pupil dynamics

**Fig. 1:**
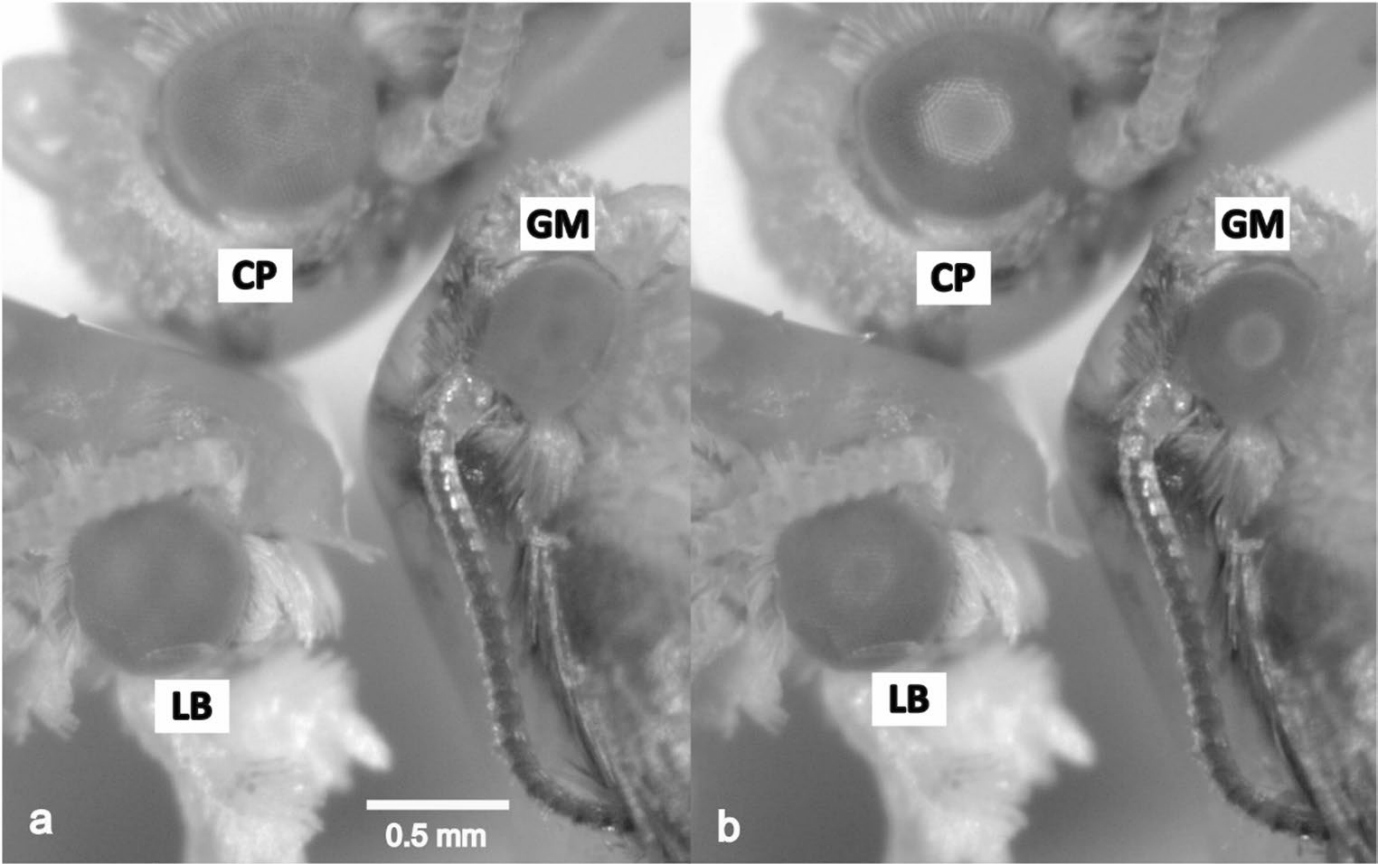
Compound eyes in three light-adapted (**a**) and dark-adapted (**b**) moths, viewed with a USB microscope and NIR illumination. Superposition pupil is the bright circle in the centre of the eye in **b**. CP, *C. pomonella*; LB, *L. botrana*; GM, *G. molesta*

The amount of light reflected by the insect eye when illuminated with an external light source served as a proxy of pupil diameter. Pupil image time series were captured with a digital camera Dino-Lite AM4115-FIT (AnMo Electronics, New Taipei City, Taiwan) and quantified with image analysis software Imagej 1.54d (Schneider et al. 2012). For each frame, a circular region of interest (ROI) encompassing the eye was manually selected, and the reflected light was estimated using the integrated density value provided by ImageJ (calculated as the product of the ROI area and the mean grey value within it). The lowest and highest recorded reflected values corresponded, in theory, to maximum and minimum pupil diameters, respectively, and these values, re-scaled to a 0 to 1 range were used to produce individual normalized pupil reflection curves. The ring illuminator, integrated in the camera, provided NIR light for pupil monitoring (λ > 800 nm, no visible pupil reaction upon illumination; light intensity could not be measured with our instrumentation) and UV light (λ ≈ 400 nm; ~8·10^16^ ph/cm^2^/s; measured with a Flame spectrophotometer (Ocean Optics, Dunedin, USA). Insects were briefly immobilized on ice and introduced head-first through the wide-opening of custom-cut pipette tips. The chilling had a minimal effect on insects, which recovered almost immediately in the pipete tip. The protruding head and prothorax were fixed to the pipette with a mixture of melted beeswax and resin, and the abdomen was allowed enough room to breathe. Three individuals were placed under the microscope, with the eye centred on the camera´s objective and light source (Fig. 1). Water-soaked paper was placed around the insects to maximize ambient humidity and slow down specimen deterioration. The setup was packed in a custom dark box located inside a temperature-controlled incubator (IPP 300, Memmert, Germany).

#### Innate pupil rhythm

In order to determine the optimal time for testing induced pupil dynamics (see below), we first examined if the eyes exhibited an internal pupil rhythm in constant darkness (i.e., independent of the external light conditions). Newly emerged adults (0- to 2-days old) were maintained under a 12:12 photoregime at 22 ± 1 °C for three days. Before the scotophase of the third day (when they were 3- to 5-days old) they were prepared for recording, and were recorded continuously (1 frame/5 min) under constant dark conditions (only NIR illumination) for 36 h (at this point, without drinking water, they started to deteriorate, Amat et al. 2022). Five individuals per species were recorded, about half males and half females.

#### Induced pupil dynamics

As the insects showed an innate circadian rhythm of pupil activity (see results), we decided to study the evoked pupil closure during the scotophase. Newly emerged adults (0- to 2-days old) were maintained under a 12:12 photoregime at either 15 ± 1 °C or 22 ± 1 °C for three days. Right before the onset of the scotophase of the 3rd day (when 3- to 5-days-old) they were immobilized in the pipettes, placed under the microscope and observed (not recorded) in darkness under the NIR light until the pupils were wide open. At this point the UV light was turned on (the NIR light was not turned off) and they were recorded (1 frame/second) until the pupils closed (it took approx. 15 min). At this point the UV light was turned off and the eyes were recorded (1 frame/minute) while the pupils gradually opened, up to a point where they did not open any more (~ 90 min). Ten individuals of each species were tested at each of the two temperature regimes, 4 to 6 of each sex.

#### Modelling pupil dynamic curves

In order to perform quantitative comparisons among the main independent variables (species, sex and temperature) of the induced pupil opening and closing curves, we first corrected the grey values obtained from the images, accounting for the camera’s non-linearity. For this purpose, we characterized the camera’s grey intensity response by photographing a calibrated ColorChecker Passport (X-Rite, Grand Rapids, USA) and fitting the resulting data to a gamma function of the form: $\:{Y}_{out}=A \cdot {{Y}_{in}}^{\gamma\:}$. This yielded the parameters A = 16.71 and γ = 0.58. We then applied the inverse of this function to correct all data points: $\:{Y}_{corr}={\left(\frac{{Y}_{meas}}{A}\right)}^{{{\upgamma\:}}^{-1}}$ where $\:{Y}_{meas}$ is the measured grey value, and $\:{Y}_{corr}$ is the corrected value. Following this correction, we normalized each curve’s minimum and maximum brightness values (originally on ImageJ’s arbitrary scale) to a standardized scale ranging from 0 to 1. Then we fitted a non-linear equation to each curve, and from these equations we obtained the slope (*b*) and the time at half intensity (half pupil brightness, *t*_50_), which were used as dependent variables in the statistical analysis (see below).

The fitted log-logistic functions had the following form:

$$\:f\left(x\right)=\frac{1}{1+\mathrm{e}\mathrm{x}\mathrm{p}\left(b\right(\mathrm{log}\left(x\right)-\mathrm{log}\left({t}_{50}\right))}$$

This function is equivalent to the Lipetz equation when *b* < 0 (pupil closing) and 1 - Lipetz equation when *b* > 0 (pupil opening) (Lipetz 1971). Parameter *b* is the slope of the linear part of the sigmoid, parameter *t*_50_ is the *x* value (time) at which *f*(*x*) (pupil brightness) is at half of its maximum value. For pupil closing curves, *b* > 0 and for opening curves, *b* < 0. In both cases, parameter *t*_50_ is a positive real number. The curves were forced to pass through origin (0,0).

### Statistical analyses

Statistical analyses were run in R 4.1.2. software (R Core Team 2021). To analyse the external eye diameter and the parameters *b* and *t*_50_ of the induced pupil dynamic curves, we used generalized linear models (GLM) with a Gaussian family link that had sex, species and temperature as independent variables (~(sex + sp + temp)^*n*^, where *n* is the order of the best fitting model). Hierarchical model selection was used to compare models from simplest (*n* = 0) to most complex (*n* = 3) using ANOVA and the Akaike information criterion (AIC). The more complex model significantly different from the previous one that had a lower AIC was chosen. Unplanned pairwise comparisons between levels of the significant model terms were made using the package emmeans() in R (Lenth 2024).

## Results

### Eye morphology

**Fig. 2:**
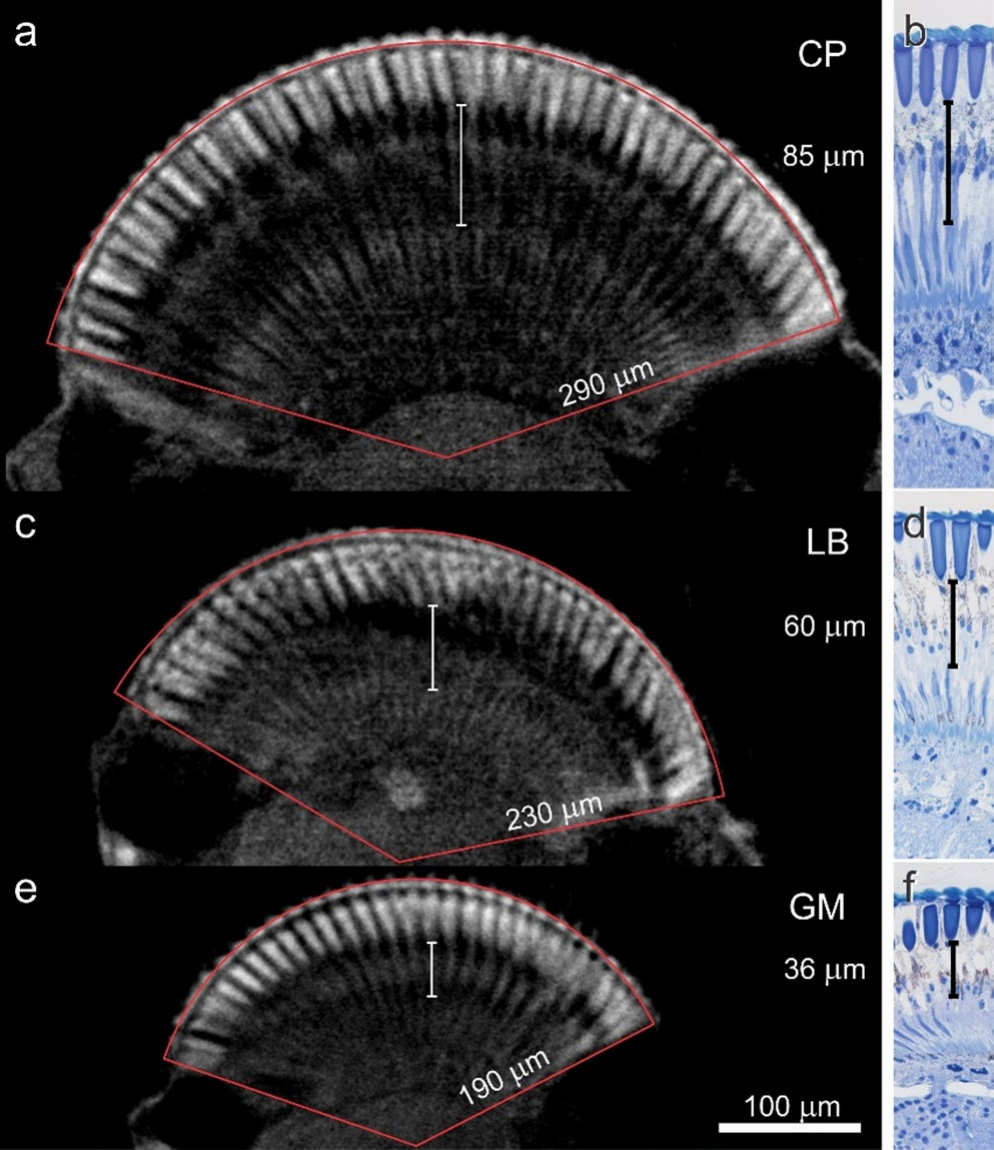
Anatomy of the eyes of *C. pomonella* (CP), *L. botrana* (LB), and *G. molesta* (GM) a-f longitudinal sections of compound eyes, imaged with micro-CT technique of intact eyes (**a**, **c**, **e**), and light microscopy of stained semithin sections (**b**, **d**, **f**). The area of the eye is delimited by a red line and the radius is indicated on one of the sides (**a**, **c**, **e**). The length of the clear zone is indicated by a vertical white scale bar (**a**, **c**, **e**), or a black scale bar (**b**, **d**, **f**)

All studied species (from the smallest to largest: GM, LB and CP (Navarro-Roldán et al. 2017) have a superposition pupil, visible after dark adaptation, composed of around 120 ommatidia, 150 ommatidia and 400 ommatidia, respectively, which can be viewed in the light-adapted and dark-adapted state with a USB microscope and NIR illumination (Fig. 1). In order to interpret pupil dynamics, the eyes were analysed with light microscopy (Fig. 2). The thickness of the clear zone was 36 µm (GM), 60 µm (LB) and 85 µm (CP), which may indicate that in the dark- and light-adapted state, the screening pigments migrate across very different lengths. Assuming equal speed of motor proteins, pupil response time should be shortest in GM and longest in CP.

Micro-CT images provided ommatidial diameter, interommatidial angle, and eye diameter (Table 1; Fig. 2) The ommatidial diameter was ~ 20 µm in LB and CP and ~ 15 µm in GM; the estimated interommatidial angle was apparently larger in the smaller moths (LB and GM) than in CP. Finally, the number of ommatidia and the eye radius correlate with the size of the individuals (Table 1; Fig. 2). In addition, in CP and LB the males have more ommatidia than females.

**Table 1:** Morphological parameters of the eyes of *C. pomonella* (CP), *G. molesta* (GM) and *L. botrana* (LB)

Species	Sex	CT-Scan, *N* = 1						Stereomicroscope, *N* = 10 (mean ± SEM)	
		Number of ommatidia	Inter-ommatidia angle	Ommatidia diameter (µm)	Eye radius (µm)	Dorso-ventral diameter (µm)	Antero-posterior diameter (µm)	Dorso-ventral diameter (µm)	Antero-posterior diameter (µm)
CP	Male	2150	3.50	20	290	660	610	632 ± 5	614 ± 5
	Female	1900	3.80	20	290	600	570	648 ± 8	604 ± 10
LB	Male	1300	4.30	20	230	480	430	492 ± 3	467 ± 4
	Female	1190	4.40	20	220	470	440	482 ± 4	461 ± 4
GM	Male	870	4.70	15	190	420	380	423 ± 7	389 ± 3
	Female	930	5.00	15	180	430	380	446 ± 6	396 ± 4

Values from CT-scans represent 1 individual of each sex and species. Values from the stereomicroscope are the mean ± SEM of 10 individuals of each sex and species

Statistical comparison of eye diameters measured with the stereo microscope on 10 males and females of each species showed that CP had significantly wider eyes than LB, and this than GM (Table 1, Supplementary Tables 1–3). In addition, sex had a minimal, but significant effect, where females had significantly wider eyes dorso-ventrally than females in CP and GM. Eye widths measured with the stereomicroscope correlated with the eye width measured with CT-scan (Pearson correlation test, *p* < 0.05), supporting the CT-scan results, which were based in just one individual of each sex and species.

### Pupil dynamics.

**Fig. 3:**
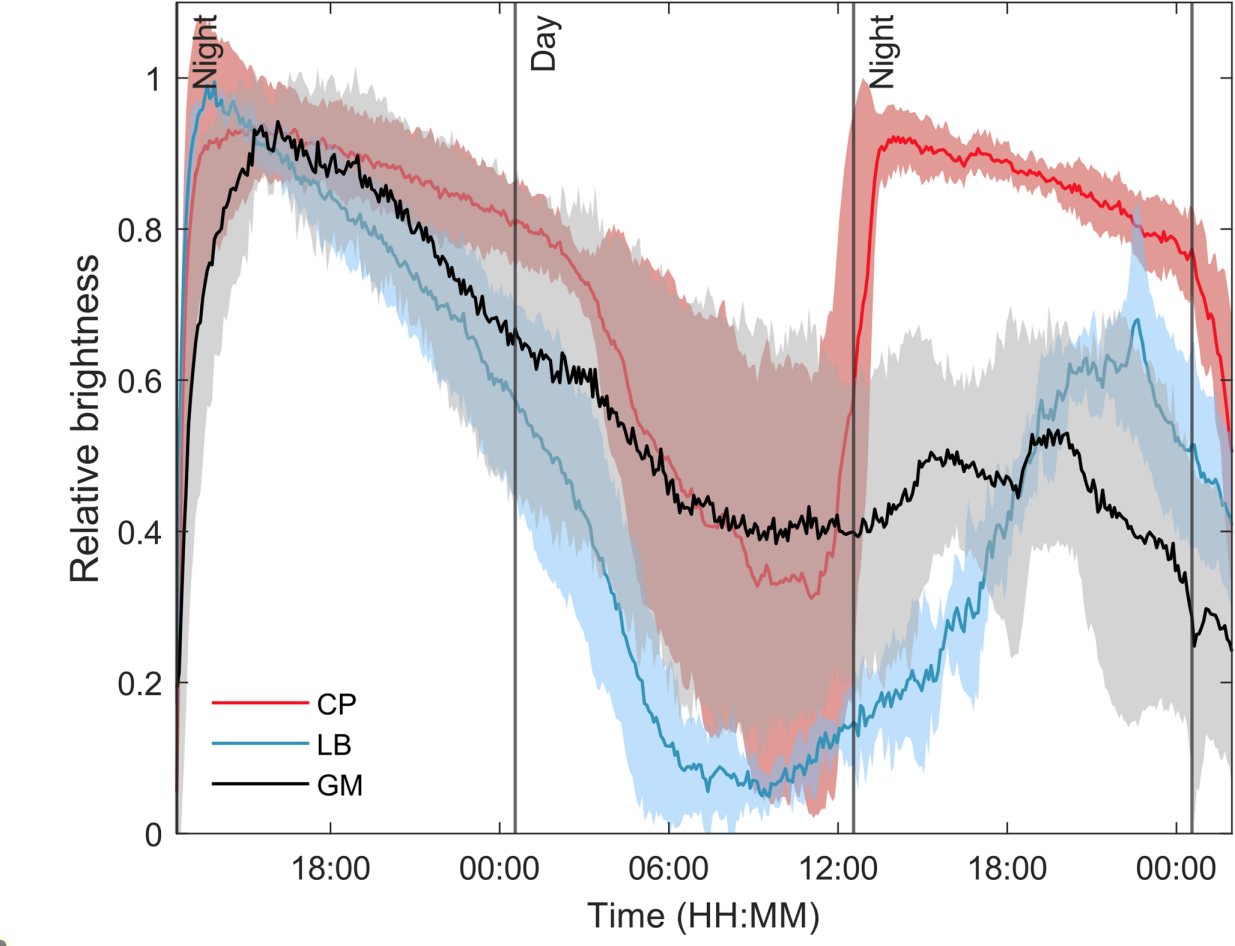
Intrinsic pupil activity during a 36-h continuous dark period after 3 d under a 12:12 L: D photoregime and constant temperature of 22 °C for the moths *C. pomonella* (CP), *G. molesta* (GM) and *L. botrana* (LB). The traces (line = mean, shaded area = ± SEM, *N* = 5 individuals per species, 50% approx. each sex) show relative pupil brightness as a function of time. Vertical lines mark the transition between the corresponding day and night of the preceding training photoregime

In order to perform the experiments during the subjective dark phase, the moths were entrained to a 12:12 h L: D photoregime and the pupil was then monitored during the following 36 h in total darkness. As expected (Nordstrom and Warrant 2000), the pupil showed strong intrinsic rhythm and spontaneously switched between the open and closed state every 12 h for up to 48 h (Fig. 3, raw data is shown in Supplementary Fig. 1). In addition to the observed circadian rhythm, the pupil gradually and irreversibly closed over time in the smallest GM and to a smaller degree in the intermediately sized LB, probably due to the deterioration of the small preparations. Further experiments were performed during the subjective night.

**Fig. 4:**
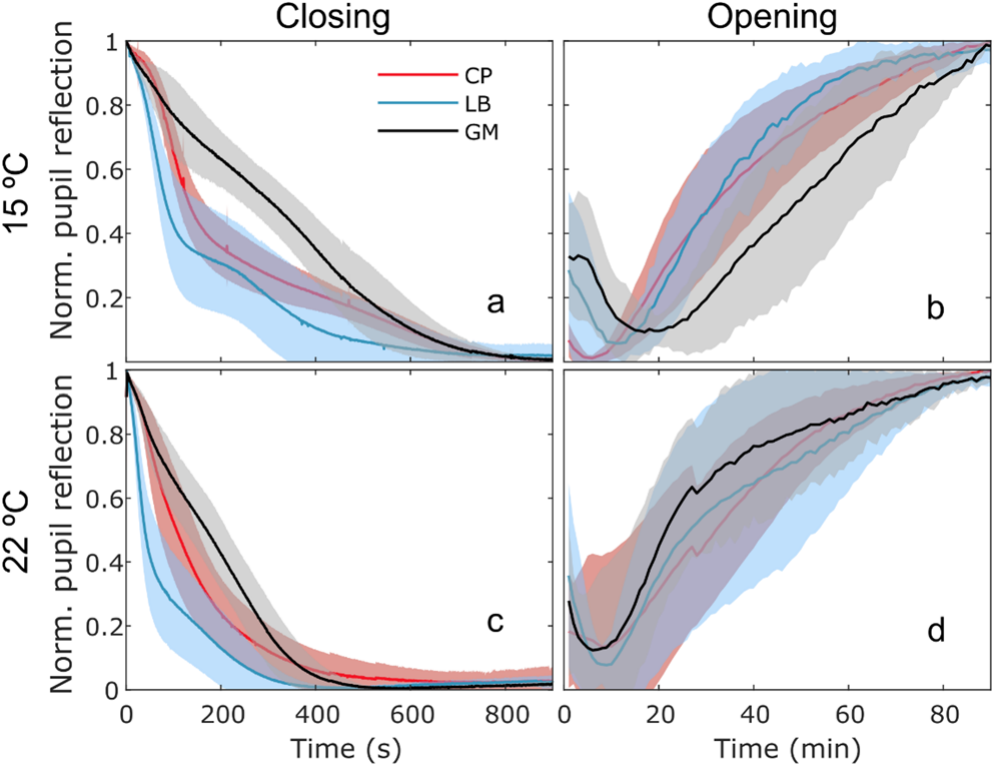
Induced pupil dynamics under (**b**, **d**) dark and (**a**, **c**) UV light adaptation at (**a**, **b**) 15 °C and (**c**, **d**) 22 °C for the moths *C. pomonella* (CP), *G. molesta* (GM) and *L. botrana* (LB). The traces (line is the mean, shaded area is ± SEM, *N* = 10 individuals per species, 50% approx. each sex) show normalized pupil reflection as a function of time

Pupil closing was induced under UV light for 15 min during the scotophase of 12:12-h L: D-trained individuals, and subsequent pupil opening was monitored right afterwards, by turning off the UV light, until the pupils were completely open. Average induced pupil closing and opening curves by temperature are shown in Fig. 4 (sexes were pooled for the sake of clarity). Individual curves were fitted to log-logistic models (individual variability and curve fit shown in Supplementary Figs. 2 and 3). For pupil closing curves the mean ± SEM goodness of fit was R^2^ = 0.96 ± 0.03, ranging between 0.87 and 0.99, and for pupil opening curves the mean ± SEM goodness of fit was R^2^ = 0.98 ± 0.02, ranging between 0.90 and 0.99, indicating in general a good model fit.

The induced pupil opening curves, which started right after the UV light was turned off, had a transient 5–15 min “bump” right at the beginning of the recording, corresponding to a sudden increase in eye brightness which quickly decreased to a minimum low before it gradually increased in a sigmoid-fashion to its maximum high at about 90 min (Fig. 4b, d). We did not observe this phenomenon in the internal rhythm recordings (Fig. 3), so it has likely been caused by the preceding exposure to UV light. The initial transient was excluded from analyses and pupil opening was further quantified by extracting the parameters of the sigmoid-shaped rising part of the curve, which started when the pupil was at its lowest brightness, right after the “bump”. The mean time at which the pupil opening started (i.e., the “bump” duration in seconds) was (mean ± SEM) 5.2 ± 2.0 s and 8.3 ± 4.8 s for CP, 19.5 ± 9.1 s and 8.1 ± 4.1 s for GM and 12.0 ± 3.8 s and 9.7 ± 3.9 s for LB, at 15°C and 22 °C, respectively (males and females pooled).

**Fig. 5:**
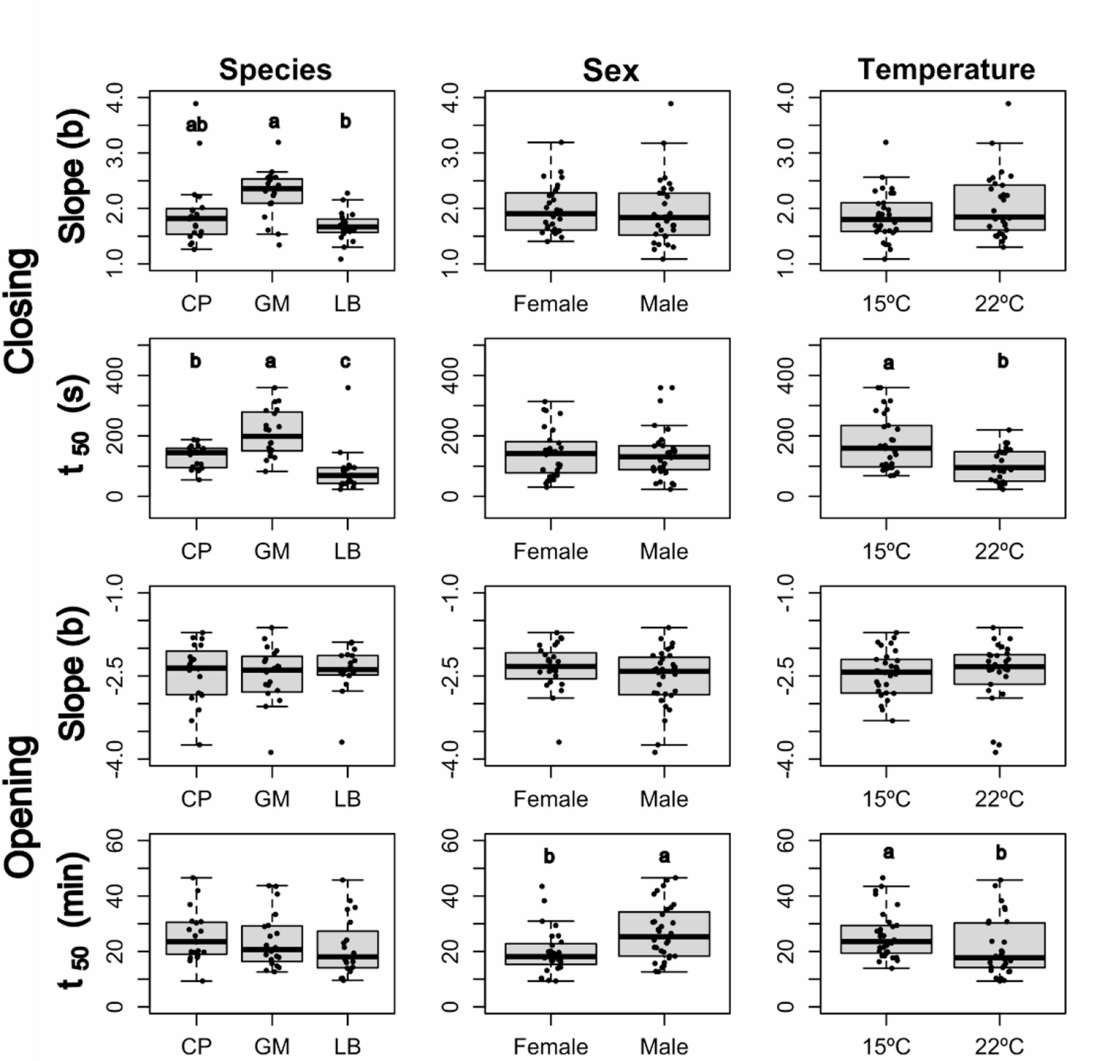
Effect of species (*C. pomonella*, CM; *G. molesta*, GM, and *L. botrana*, LB), sex and temperature on the slope (*b*) and time to half opening or closing (*t*_50_) of the time course of light- and dark adaptation of the eyes. Within each plot, different letters among treatment levels indicate significant differences (Tukey´s test, *P* < 0.05). Box plots show median (horizontal line), first and third quartile (box) and 1.5*inter-quartile range (vertical bars)

Statistical analysis of the estimated slopes (*b*) and half-opening values (*t*_50_) from these curves revealed significant species, sex and temperature effects (Supplementary Tables 4–6 and Fig. 5). LB closed its pupils faster (i.e., lower t_50_) than the other two species, regardless of the temperature, but the closing slope was similar to that of CP. On the other hand, GM had the highest closing slope *b* (albeit not significantly different from CP), but also the highest *t*_50_, meaning that its pupil takes longer to close in a significant way but the closing speed is more constant over time, making it the slowest at low temperature, and almost as slow as CP at higher temperatures. Higher temperature significantly shortened half-closure time. Regarding pupil opening, no difference across species was observed in the slope or half-opening time, but females reached half-opening time significantly earlier than males, and higher temperature significantly shortened half-opening time (Fig. 5, Supplementary Table 6).

## Discussion

We have found that pupil dynamics differ significantly among the three differently sized tortricid moths. Furthermore, temperature affects pupil dynamics differently in each species. Eye size in these small microlepidoptera, as estimated by eye radius *r*, is close to (230 and 290 µm in LB and CP) or below (190 μm in GM) the proposed theoretical minimal size for a functional optical superposition insect eye (*r* > 250 µm; Meyer-Rochow and Gál 2004). In spite of the calculated lower limit, the superposition pupil is well resolved even in the smallest species, GM (Fig. 1), indicating that further theoretical and experimental work is needed to fully understand optical superposition eyes. All three species have functional superposition optics, but because pupil aperture decreases with decreasing eye size, the species with the smallest superposition aperture should have the lowest light sensitivity, following the sensitivity formula of Warrant and Nilsson (1998), which is based on anatomical parameters, assuming that, in our case, photoreceptor diameter and photoreceptor length also get smaller with eye size. Accordingly, GM might have the lowest sensitivity of the three species, which is in agreement with its diurnal life style, while the intermediate and highest sensitivity could be expected in the crepuscular and nocturnal species LB and CP, respectively.

Sex differences in eye size, although statistically significant, were minimal (less than 0.5% of the total ANOVA model variance) and contributed only slightly to differences in pupil dynamics between sexes (females, having smaller eyes than males, reached half-time opening earlier than them). Females had larger dorso-ventral eye diameters than males in CP and GM, which is in accordance with the bigger size of females, but not in the crepuscular moth species LB. Pupil dynamics was controlled by the intrinsic circadian rhythms in the three tortricids. Circadian rhythmicity of eye pigments was already shown in CP (Nordstrom and Warrant 2000), but not in the other two species. Goldsmith and Bernard (1974) reviewed daily rhythms of pigment position and showed that many insects preserve it under constant illumination conditions, but in some species such as the Pyralid moth *Ephestia kuehniella* (Zeller) there is no circadian rhythm (Day 1941). The beetle *Tenebrio molitor L.* (Ro and Nilsson 1993) and the bedbug *Triatoma infestans* Klug (Reisenman et al. 2002) also show circadian rhythms of pigment migration under constant dark or light conditions. External temperature does not affect screening pigment movement in *T. infestans* (Lazzari et al. 2011), whereas in CP exceeding a threshold temperature induces a fast migration to the light-adapted position regardless of external light conditions (Nordström and Warrant 2000). This suggests that, in some species, temperature could be an important factor for triggering pupil dynamics, due to temperature differences between the day and the night, which may be used by an organism to indirectly estimate the light conditions, usually associated with a given temperature range.

Circadian rhythm observations showed that in the tortricids species opening and closing have a similar duration under constant illumination conditions and, in fact, our curve for CP is very similar to the one reported by Nordström and Warrant (2000) for this species. Ro and Nilsson (1993) also reported similar durations for pupil opening and closing of a beetle species during constant light and constant dark conditions, but there are not many references comparing this parameter under constant illumination conditions. We observed that induced pupil closing was noticeably shorter than induced pupil opening, whereas the duration of these two periods was similar in insects maintained under constant darkness. Dreisig (1981) demonstrated that the position and speed of screening pigments depend solely on the absolute illumination level, producing similar movement curves at both dawn and dusk. However, other studies—such as Bernard et al. (1984) on pyralids; Stavenga et al. (1977) on butterflies; and Nordtug (1990) and Nordtug and Melo (1992) on noctuids—reported significantly faster closing curves compared to opening ones. These discrepancies likely arise from the highly variable experimental conditions, as each study employed stimulus lights with substantially different wavelengths and intensities—the latter often differing by several orders of magnitude.

Therefore, it is plausible that an intense light stimulus accelerates the closing process to prevent photoreceptor saturation and protect the insect from temporary blindness. Alternatively, the observed asymmetry may result from the abrupt and artificially intense change in illumination during the experiment, or due to the use of monochromatic UV light for stimulation, which could have caused microvillar membrane degradation (Langer et al. 1986), or the accumulation of long-lived photo-products, such as the metarhodopsin isoform, which can potentially trigger the pupil closure until enzymatic degradation (Stavenga and Hardie 2011). We note that we deliberately used the integrated ring illuminator LEDs for both monitoring and stimulation, to demonstrate the applicability of a commercial-grade USB microscope, and to keep the set-up small, so that it could be placed within the climatized incubator. The chosen stimulation wavelength sufficiently overlaps with the sensitivity of all photoreceptor classes, which is expected in Tortricidae (Martin-Gabarella et al. 2023). Thus, we demonstrate that a simple, off-the shelf device could be used in future studies for monitoring the effects of environmental pollution in insects with superposition eyes. However, to properly measure the pupil action spectrum, an epi-illumination optical pathway with a beam splitter would be required.

To investigate interspecific differences in pupil closure dynamics, we compared the temporal response profiles of the three studied species by inducing pupil closure with UV light **(**Fig. 4a and c**)**. All curves have a brief initial delay of 2–4 s before the onset of the closing process (not shown in the figure). In LB and CP, this is followed by a rapid decline in pupil brightness that decelerates progressively. In contrast, GM displays a more gradual and uniform reduction throughout the closing period. LB and CP exhibit a bimodal pattern—an inflection or plateau—that becomes more pronounced at lower temperatures. A similar curve shape was described by White et al. (1983) in the moth *Manduca sexta* (L.) (Lepidoptera: Sphingidae), though without considering temperature or offering a mechanistic explanation. Bernard et al. (1984) described a triphasic reflectance response in the moth *Amyelois transitella* (Walker) (Lepidoptera: Pyralidae), consisting of an initial slight decrease due to pigment migration from photoreceptors, a transient increase caused by pigment congregation at the distal tip of the rhabdom, and a final sustained decline as pigments accumulate from both photoreceptors and accessory pigment cells. Nordtug (1990) later reported that noctuids exhibit only the second and third phases. In our data, the closure curves appear to begin directly at the third phase, with no detectable evidence of the initial two. Thus, the bimodal features observed in LB and CP do not align with the triphasic pattern previously described. We assume that in small superposition eyes, the different phases of light adaptation overlap in time and can’t be separated without further experimental manipulation. In our study, LB had the fastest closing speed (lowest *t*₅₀). GM, however, had the steepest slope (*b*), reflecting its steady, linear-like closure. The lower *b* values in LB and CP likely result from the mid-curve inflection, which reduces overall steepness.

The rapid dynamics observed in LB are consistent with its ecological context, as it is active during periods characterized by abrupt changes in ambient light. Such conditions likely favour rapid pupil adjustments to maintain visual performance (Warrant and Somanathan 2022). Conversely, GM—despite having the smallest eyes and presumably the shortest pigment migration paths—displays the slowest yet most consistent closure kinetics, preserving the shape of the closing curve to a greater extent than other species. Temperature was found to prolong closure time in all three species; however, GM exhibited the strongest temperature dependence, with closure duration increasing more markedly as temperature declined. This raises the possibility that thermal sensitivity of pupil dynamics may depend by morphological traits such as eye size. After the “bump”, statistical analysis reveals no significant differences among species—unlike the closing phase, where interspecific variation was evident. This suggests that the opening response in darkness is less species-specific and less finely tuned than the closing mechanism, and is also uniformly slowed by lower temperatures. The observed effects of sex remain unexplained.

The main interspecific differences lie in the closing process. LB, a crepuscular species, closes its pupil fastest, with dynamics similar to the nocturnal CP, which is bigger and slower. In contrast, the diurnal and smallest species, GM, shows distinct closure dynamics and greater temperature sensitivity. This may relate to its smaller size, which can increase heat loss (Kozlowski et al. 2004), or to diurnal activity, which provides less natural light variation but higher photon availability. Consequently, GM may rely on alternative protective strategies, such as lower visual sensitivity or adaptations typical of diurnal species with apposition eyes (Warrant and McIntyre 1996). Our hypothesis that pupil speed is inversely proportional to eye size has been therefore disproved. The similarity in the sigmoid portion of the opening curves across species may result from all experiencing a drop below their respective light thresholds for pupil opening (Dresig 1981). However, the abrupt light-off in our experiment likely masked any interspecific threshold differences.

In summary, our results highlight clear interspecific differences in pupil dynamics, with LB exhibiting the fastest and most responsive closure, likely reflecting adaptation to environments with rapid light fluctuations. In contrast, GM displays the slowest and most thermally sensitive response, with a markedly different, more uniform closure profile. These distinct dynamics may be linked to differences in ecological niches, visual ecology, or eye morphology, suggesting divergent evolutionary pressures shaping the pupil mechanisms across species.

## Supplementary Information

Below is the link to the electronic supplementary material.

Supplementary Material 1

## Data Availability

Raw data and R-scripts are publicly available in the repository at the University of Lleida (https://doi.org/10.34810/data2466).
