# Supplementary material for: Pupil dynamics reveal the tuning of tortricid moths to diel activity
Source: J Comp Physiol A Neuroethol Sens Neural Behav Physiol. 2025 Oct 9;212(2):271–81. doi: 10.1007/s00359-025-01759-0 (PMC13086707; doi:10.1007/s00359-025-01759-0)
Supplement: Supplementary file 1 — Supplementary Material 1 [file 359_2025_1759_MOESM1_ESM.docx]

**Pupil dynamics reveal the tuning of tortricid moths to diel activity**

Alejandro Martín-Gabarrella, César Gemeno, Aleš Škorjanc, Andrej Meglič and Gregor Belušič

**Supplementary material**

**Supplementary Table 1**. Comparison of GLM models analyzing the effect of species and sex on the external eye diameter of *C. pomonella*, *G. molesta* and *L. botrana*. N= 10 individuals per species (approx. 50% each sex).

**Supplementary Table 2**. Summary of GLM models analyzing the effect of species and sex on the external eye diameter of *C. pomonella*, *G. molesta* and *L. botrana*. N= 10 individuals per species (approx. 50% each sex).

**Supplementary Table 3**. Pairwise comparison of the significant terms of the GLM models analyzing the effect of species and sex on the external eye diameter (in mm) of *C. pomonella* (CP), *G. molesta*, (GM), and *L. botrana* (LB). N= 10 individuals per species (approx. 50% each sex). A) Effect of sex and species (significant in both, antero-posterior and dorso-ventral aspects). B) Effect of species with sex and sex within species (significant species*sex effect, only in the dorso-ventral aspect)

**Supplementary Table 4**. Comparison of GLM models analyzing the effect of species, sex and temperature on the pupil opening and closing curve parameters (slope b, and time to half brightness t_50_) of dark and light adapted eyes of *C. pomonella*, *G. molesta* and *L. botrana*. Models were compared with ANOVA and the Akaike information criterion (AIC). N= 10 individuals per species (approx. 50% each sex).

**Supplementary Table 5**. Summary of the selected GLM models analyzing the effect of species, sex and temperature on the pupil opening and closing curve parameters (slope b, and time to half brightness t_50_) of dark and light adapted eyes of *C. pomonella*, *G. molesta* and *L. botrana*. N= 10 individuals per species (approx. 50% each sex).

**Supplementary Table 6**. Pairwise comparison of the selected GLM models analyzing the effect of species, sex and temperature on the pupil opening and closing curve parameters (slope b, and time to half brightness t_50_) of dark and light adapted eyes of *C. pomonella* (CP), *G. molesta* (GM), and *L. botrana* (LB). N= 10 individuals per species (approx. 50% each sex).

**
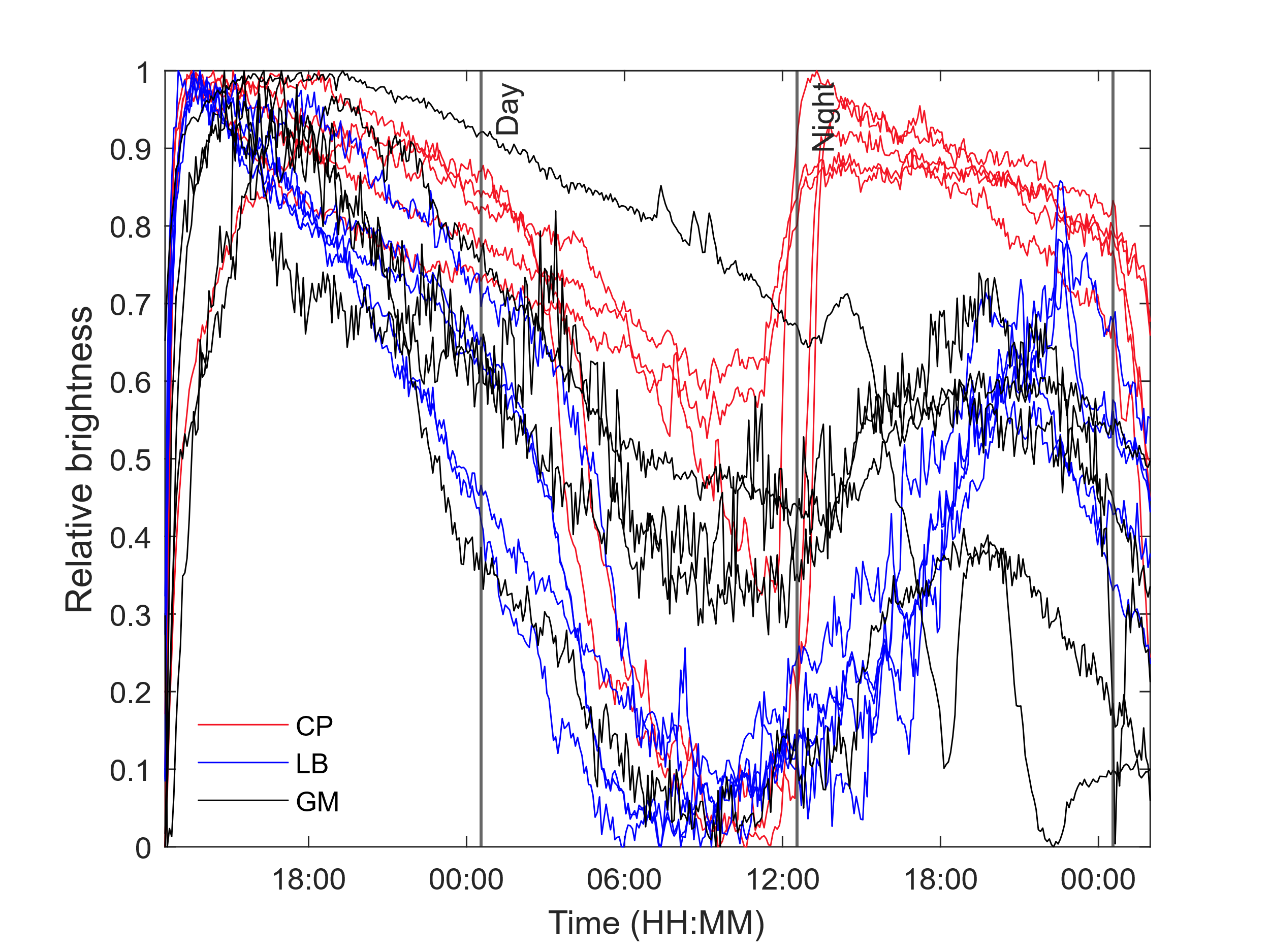
**

**Supplementary Fig. 1**. Intrinsic pupil activity as a function of time during a 36 h continuous dark period after 3 d under a 12:12 L:D photoregime for the moths *C. pomonella* (CP), *G. molesta* (GM) and *L. botrana* (LB). Each line is a different individual (N=5 individuals per species, approx. 50% each sex). Vertical lines mark the transition between day and night of the corresponding previous training photoregime.


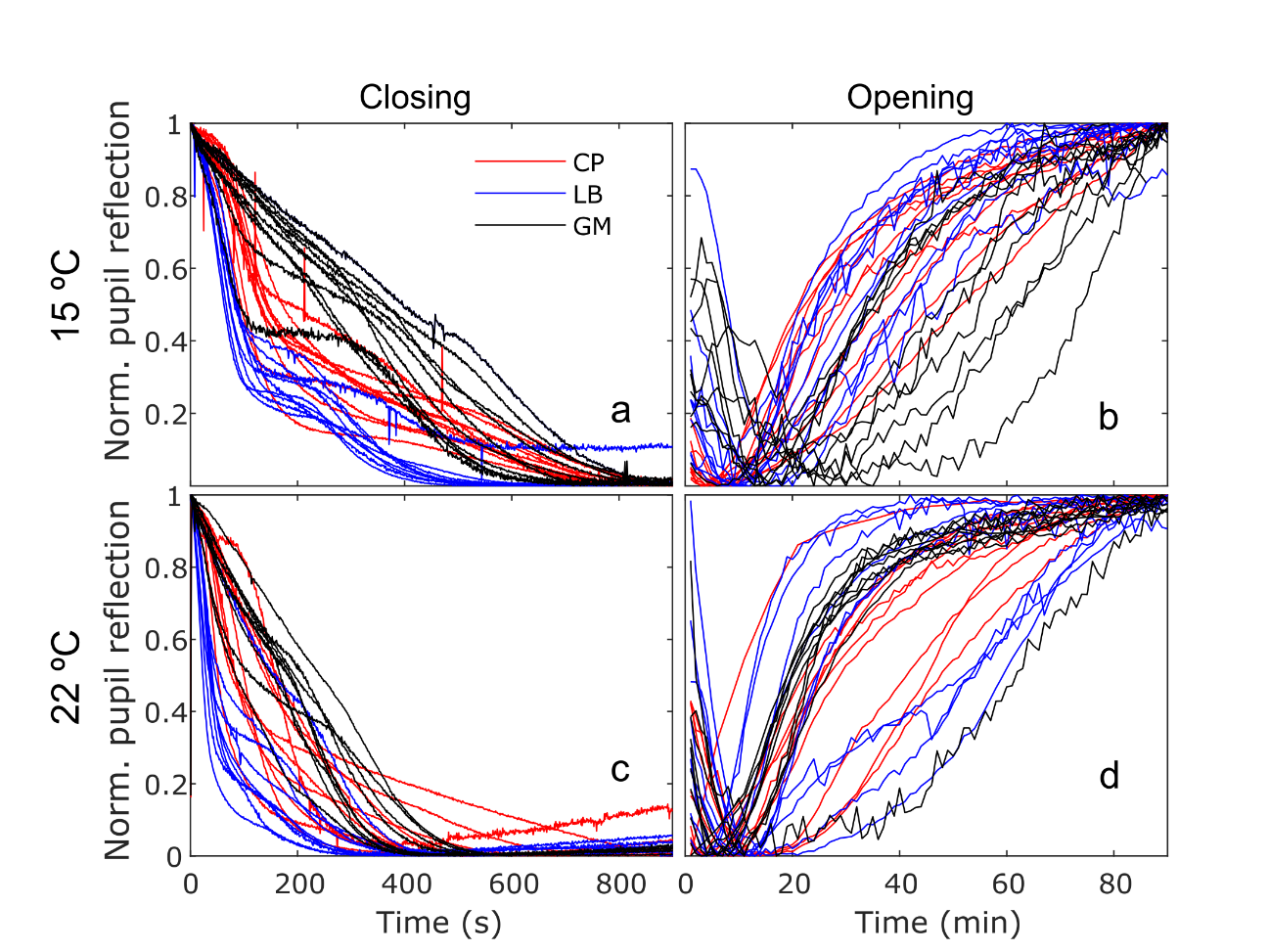


**Supplementary Fig. 2**. Induced pupil dynamics under (b, d) dark and (a, c) UV light adaptation at (a, b) 15°C and (c, d) 22°C for the moths *C. pomonella* (CP), *G. molesta* (GM) and *L. botrana* (LB). Each line represents the normalized pupil reflection as a function of time of a different individual. N= 10 individuals per species, with approximately 50% of each sex. For the sake of clarity males and females are not labelled differently.


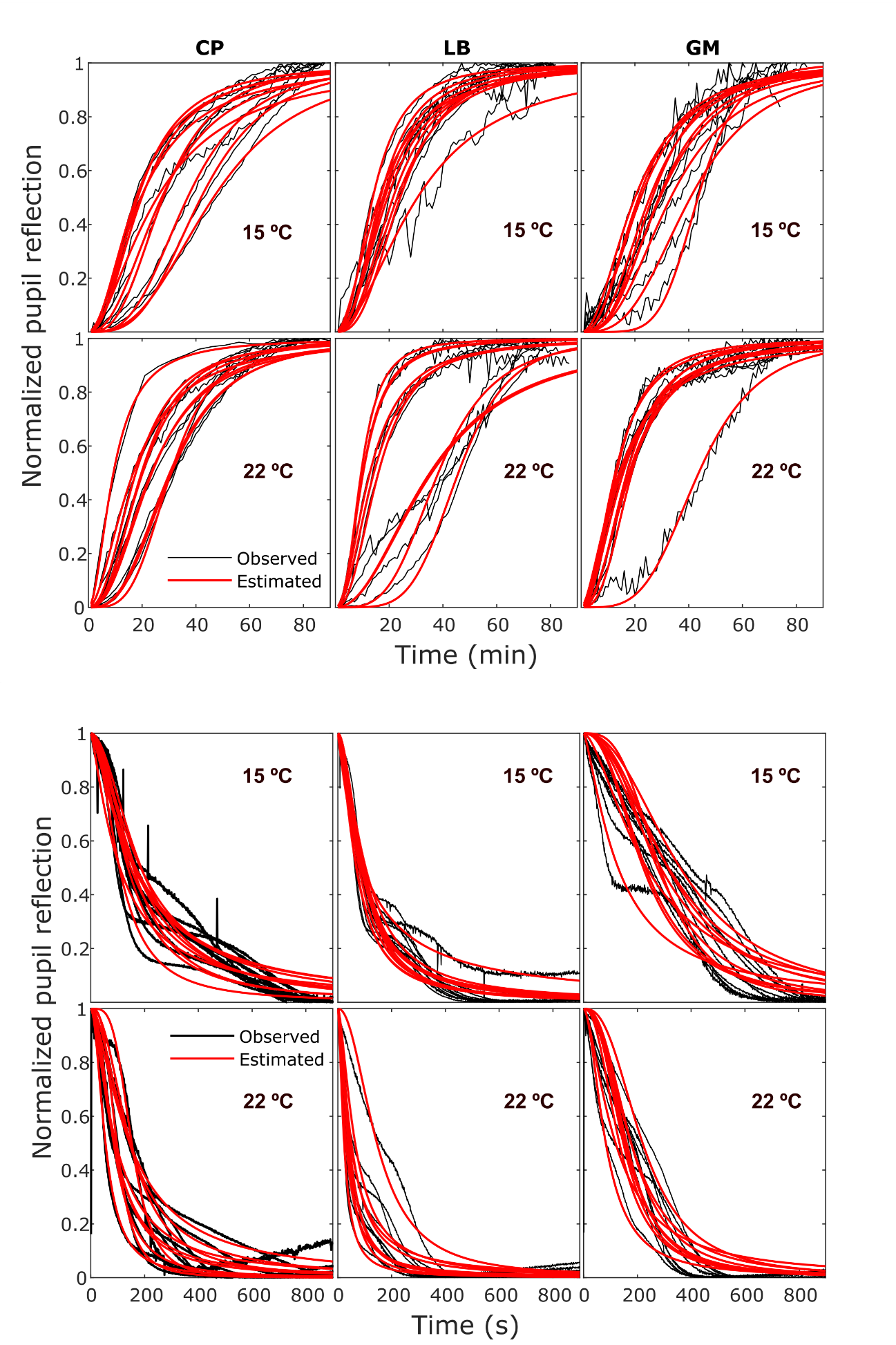


**Supplementary Fig. 3**. Overlaying of the estimated values from the log-logistic models (red lines) on the observed values (black lines) to illustrate model fit of induced pupil opening and closing at two different temperatures (15°C and 22°C) for the moths *C. pomonella* (CP), *G. molesta* (GM) and *L. botrana* (GM). N=10 individuals per species, approximately 50% of each sex. Males and females are not labelled differently for the sake of clarity.
